# Antimicrobial Activity of Chitosan-Based Films Enriched with Green Tea Extracts on Murine Norovirus, *Escherichia coli*, and *Listeria innocua*

**DOI:** 10.1155/2020/3941924

**Published:** 2020-07-02

**Authors:** Collins Amankwaah, Jianrong Li, Jaesung Lee, Melvin A. Pascall

**Affiliations:** ^1^Department of Food Science and Technology, The Ohio State University, 2015 Fyffe Road, Columbus, Ohio, USA; ^2^Department of Veterinary Biosciences, The Ohio State University, 1925 Coffey Road, Columbus, Ohio, USA

## Abstract

Edible films can be designed to serve as carriers of antimicrobial agents and be used to control pathogenic foodborne viruses and bacteria. This research tested this concept by dissolving green tea extract (GTE) in chitosan film-forming solutions (FFS) and using it to prepare dried chitosan edible films. As a control, the GTE was also dissolved in deionized water (DW). The FFS and the dried chitosan films with the GTE and the DW without chitosan were all evaluated against murine norovirus (MNV-1), *Escherichia coli* K12, and *Listeria innocua*. Both the FFS and the DW with GTE were incubated with ~10^7^ PFU/ml of the virus suspensions for 3 h. The chitosan films with GTE were incubated for 4 and 24 h at 23 ± 1°C. The results showed that the DW containing 1, 1.5, and 2.5% aqueous GTE, significantly (*p* < 0.05) reduced MNV-1 plaques by 1.7, 2.5, and 3.3 logs after 3 h exposure, respectively. Similarly, FFS containing 2.5 and 5.0% GTE reduced MNV-1 counts by 2.5 and 4.0 logs, respectively, after 3 h exposure. The dried chitosan films with 5, 10, and 15% GTE were also effective against MNV-1 infectivity. After 24 h incubation, the 5 and 10% chitosan GTE films produced significant (*p* < 0.05) titer reductions of 1.6 and 4.5 logs, respectively. Chitosan films containing 15% GTE reduced MNV-1 plaques to undetectable levels in 24 h. All chitosan GTE films reduced *E*. *coli* K12 and *L*. *innocua* populations to undetectable levels in tryptic soy broth after 24 h exposure. The results of this study showed that edible films enriched with GTE have potential to reduce both foodborne viruses and bacteria.

## 1. Introduction

Foodborne illnesses caused by bacteria and viruses have emerged as a major worldwide public health concern. In the United States, an estimated 48 million people suffer from foodborne diseases every year [1]. From this number, 128,000 are hospitalized and 3,000 die. The human norovirus alone accounts for about 21 million cases. This makes the virus the leading cause of gastroenteritis in the United States. The virus is normally transmitted via fecal/oral routes, by the ingestion of contaminated water and food, from persons to person, and from vomitus and aerosols [2]. The seriousness of a norovirus outbreak is further heightened by its ability to persist in the environment and especially on food contact surfaces. It also has a low infection dose (10-100 virions), and large numbers of virions are shed in the feces of infected individuals [3]. If contaminated, common risk foods associated with outbreaks include minimally processed and ready-to-eat commodities such as berries and oysters. These foods could become cross-contaminated by careless food handlers and/or by contaminated and unsanitary water at the preharvest or postharvest stages. In addition to noroviruses, outbreaks from bacterial pathogens such as *Escherichia coli* O157:H7 and *Listeria monocytogenes* have created a need for adequate control measures while maintaining the quality of ready-to-eat and processed foods.

An active food package (such as antimicrobial packaging) can serve as a nonthermal tool for controlling foodborne pathogens. Mehyar and Holley [4] defined active packaging as one intended to sense an internal or external environmental change and respond by changing its own properties or its own internal environment. In antimicrobial packaging, preservatives are added to the material either during the film synthesis process or as a coating on the surface of the material after the extrusion process. Examples of these can be seen in reports from several researchers who have used edible films impregnated with antimicrobial agents designed to minimize bacterial growth on selected ready-to-eat foods [5, 6]. Additionally, interest is increasing in the use of naturally derived antimicrobial agents for the development of active antimicrobial packages because these are perceived to be safer than synthetic chemicals [7, 8].

Chitosan has been extensively studied for its dried film-forming ability and its inherent antimicrobial activity. Chitosan is derived from chitin, the second most abundant polysaccharide after cellulose [9]. Therefore, a chitosan-based film can be a suitable low-cost candidate as a carrier for plant-based antimicrobial compounds. However, chitosan has limited solubility in water, and to make film-forming solutions, it must be dissolved in an acid. Also, the choice of other antimicrobial compounds for blending with chitosan must be carefully selected because previous studies have shown that some compounds could negatively affect the antimicrobial activity of chitosan itself [10, 11]. Indeed, selected plant extracts have been shown to have broad spectral activities against bacteria, fungi, and viruses. For example, polyphenolic compounds such as catechins and polyphenons, found in green tea extracts (GTE), exhibited strong antiviral activities against foodborne viruses [12, 13]. Falcó et al. [14] also reported on the antiviral activities of GTE in an edible coating solution against MNV-1. However, a search of the literature shows that little is reported on the use of dried edible films as carriers for plant-based compounds having antiviral efficacy.

Human norovirus studies have been limited by the fact that the virus lacks a robust reproducible in vitro cultivation system. Recent findings have shown that human norovirus can be grown in human B cells in the presence of enteric bacteria [15]. In addition, Ettayebi et al. [16] found that human norovirus can be cultivated in stem cell-derived human enteroids. However, the robustness of these culture systems for food safety research has not been evaluated. Therefore, murine norovirus (MNV-1), which has similar properties to human norovirus, was used in this present study as a surrogate [17].

The objective of this study was to investigate the antiviral activity of a chitosan-based film against foodborne microorganisms in a food simulant containing green tea extract (GTE). This study adds to the body of published reports on the use of edible materials as tools for antiviral and antibacterial packaging of foods susceptible to microbial contamination.

## 2. Materials and Methods

### 2.1. Film Materials and Preparation

Medium molecular weight chitosan powder (94% purity) was obtained from Huantai Goldenlake Carapace Products Co., Ltd. (Qingdao, China) and used as the base material to form the test films. Glycerol was obtained from Fisher Scientific (Fisher Scientific, Fair Lawn, NJ, USA) and used in the film as a plasticizer. Glacial acetic acid was purchased from J.T. Baker (Phillipsburg, NJ, USA) and used as a solvent for the film-forming compounds. The green tea extract (GTE), soluble in water, with an EGCG content of 50% was purchased from Herb Store USA (Walnut, CA, USA) and used in this study.

**Figure 1:**
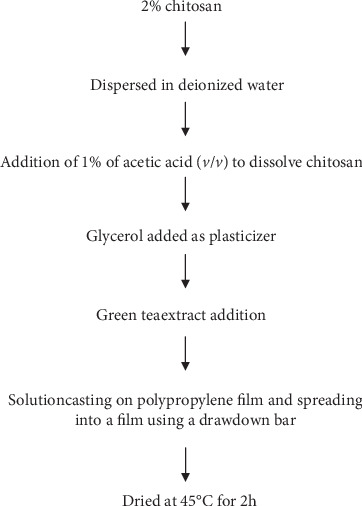
Flow chart of chitosan/GTE blend and film formation.

To make the films, chitosan (2% *w*/*w*) was dissolved in a 1% acetic acid solution with glycerol (Figure 1). Green tea powder at different concentrations (0-15%) was then added to chitosan film-forming solutions (FFS) as depicted in Table 1. The solutions were stirred using a magnetic stirrer for 30 min to dissolve the powdered extracts. The film-forming solutions were then degassed in a water bath sonicator. To form a film, a 20 g aliquot of the FFS was poured onto a Mylar (DuPont Inc., Kinston, NC, USA) polyethylene terephthalate (PET) sheet attached to a glass plate and a drawbar used to spread the solution into a thin film. After drying in an oven at 45°C for 2 h, the film was peeled off from the Mylar backing and stored in an air-tight container at 23°C for further testing.

### 2.2. Virus Stock Preparation and Plaque Assay

The murine norovirus (MNV-1) strain, used as a human norovirus surrogate, was obtained from Herbert W. Virgin IV, Washington University School of Medicine. Murine macrophage cell line RAW 264.7 (ATCC, Manassas, VA, USA) was used to grow the MNV-1. High-glucose Dulbecco's Modified Eagle Medium (DMEM) and Fetal Bovine Serum (FBS) were purchased from Invitrogen (Carlsbad, CA, USA) and also used to grow the cells. Six-well plates were obtained from Corning Life Sciences (Wilkes-Barre, PA) and used to culture the cells for the plaque assay. The MNV-1 was grown in a monolayer of RAW 264.7 cell line cultured in DMEM that was supplemented with 10% FBS at 37°C under a 5% CO_2_ atmosphere. Confluent RAW 264.7 cells were infected with MNV-1 at a multiplicity of infection (MOI) of 1.0. After 1 h of incubation at 37°C, 20 ml of serum-free DMEM was added and incubated at 37°C under 5% CO_2_. The virus was harvested after 2 days of incubation by three freeze-thaw cycles and centrifugation at 3,000 g for 20 min at 4°C. The supernatant was collected, and the viral titer determined by plaque assay.

The MNV-1 plaque assay was performed on RAW 264.7 cells seeded into six-well plates and incubated for 24 h at 37°C under a 5% CO_2_ atmosphere according to a method reported by Wobus et al. [18]. The cell monolayers were then infected with 400 *μ*l of appropriate serial dilutions of the virus suspension and the plates incubated for 1 h at 37°C, with gentle shaking every 15 min, to allow for the virus attachment. After removal of the inocula, the cells were overlaid with 2 ml of Eagle Minimum Essential Medium (MEM) containing 1% agarose, 2% FBS, 1% sodium bicarbonate, 0.1 mg of kanamycin/ml, 0.05 mg of gentamicin/ml, 15 mM HEPES (pH 7.7), and 2 mM L-glutamine (Invitrogen). After 48 h of incubation at 37°C and 5% CO_2_, the plates were fixed and stained with 10% formaldehyde and crystal violet. The plaques were then counted.

### 2.3. Antiviral Testing of GTE in Water and Film-Forming Solution

The GTE was dissolved in deionized water (pH 6) and sterilized by passing it through a 0.2 *μ*m filter. Concentrations of up to 5.0% GTE in deionized water were tested by adding equal volumes of the virus suspensions (10^7^ PFU/ml). Volumes of 0.5 ml each of the virus suspensions and the DW were mixed to give final extract concentrations of 1.0, 1.5, and 2.5%. The mixtures were incubated at 23 ± 1°C on a rotary shaker for 3 h. For the control, an equal volume of DMEM (0.5 ml) was mixed with the virus suspension. After 3 h, 0.5 ml of the treated samples and the control was withdrawn and diluted with 0.5 ml DMEM. After 10-fold serial dilutions, the appropriate solutions were plaque assayed as previously described.

A similar procedure was followed for testing reductions in the virus infectivity caused by the FFS. To perform this test, 5 and 10% GTE in FFS were mixed with equal volumes (0.5 ml) of the virus suspensions to yield final concentrations of 2.5 and 5.0% GTE in the FFS, respectively. The controls were DMEM and 2% chitosan solution individually mixed with equal volumes (0.5 ml) of the virus suspensions.

### 2.4. Antiviral Testing of GTE in Chitosan Films

In testing the virucidal activity of the dried chitosan films, the method of Haldar et al. [19] was used with slight modifications. Film samples containing different concentrations of GTE (5, 10, and 15%) and measuring 2.5 cm × 2.5 cm were individually placed at the bottom of the wells of a 6-well plate. To each sample, a 2 ml aliquot of the virus suspension (10^7^ PFU/ml) was added. These were then incubated at 23 ± 1°C in an incubator shaker for 24 h. Virus suspensions with no added chitosan film (AS) and one with 2% chitosan film but with no added GTE extract were used as controls. After 4 and 24 h, 0.5 ml of the treated samples and the controls, respectively, was withdrawn and diluted with 0.5 ml of DMEM. Ten-fold serial dilutions were then made and the appropriate solution plaques assayed as previously described.

### 2.5. Antibacterial Testing of GTE in Chitosan Films

For the bacterial testing, only the dried films were used. *Escherichia coli* K12 (ATCC 29181) and *Listeria innocua* (ATCC 33090) were purchased from the American Type Culture Collection (Manassas, VA, USA). The bacteria species were individually cultured by transferring a loopful of frozen (-80°C in 30% glycerol) *E. coli* K12 or *L. innocua* into 20 ml of sterile tryptic soy broth (TSB) (Difco, Sparks, MD, USA) with an inoculation loop. The organisms were incubated for 24 h at 37°C to reactivate them. Afterwards, a loopful of each revived bacterial suspension was transferred to separate tryptic soy agar slants and incubated for 24 h at 37°C. The slants were then refrigerated at 4°C and used as stock cultures. Before each experiment, a loopful of the respective bacteria was taken from the slants and cultured in a 20 ml sterile TSB for 24 h at 37°C. Bacterial suspensions containing approximately 10^7^ CFU/ml were prepared in TSB from the overnight culture and used for the antibacterial testing of the films.

Film samples (2.5 cm × 2.5 cm) were placed in sterile plates and 2 ml of each bacterial suspension (10^7^ CFU/ml) added. Cultures without a film and another with the 2% chitosan film, but with no added GTE extract, were used as controls. The plates were incubated and shaken at 60 rpm in an incubator shaker at the optimal temperature for 24 h. From this, the samples were taken at 0, 3, 6, 12, and 24 h intervals and diluted with 0.1% peptone solutions. Appropriate dilutions were pour plated using TSA. The plates were incubated for 36 h at 37°C and the number of colonies counted using a Darkfield plate counter (American Optical, Buffalo, NY, USA).

### 2.6. Statistical Analysis

All data were analyzed using the analysis of variance (ANOVA) and Tukey's multiple comparisons test was used to determine the antimicrobial efficacies of different concentrations of the GTE in the solutions and in the dried chitosan films. The level of significance was set at *p* < 0.05. The statistical package used in the study was IBM SPSS for Windows (Armonk, NY, USA). All data presented are the means of four replications.

## 3. Results

### 3.1. Antiviral Effects of GTE in Water and the Chitosan Film-Forming Solution

**Figure 2:**
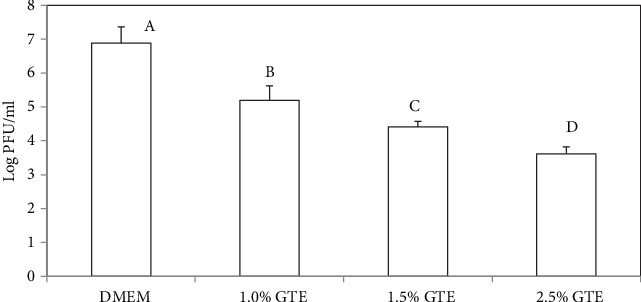
Infectivity of MNV-1 after treatment with different levels of aqueous solution of green tea extracts (GTE) and Dulbecco's Modified Eagle Medium (DMEM). Reduction in MNV-1 infectivity was detected by plaque assay after 3 h of incubation at 23 ± 1°C. Error bars indicate standard deviation.

**Figure 3:**
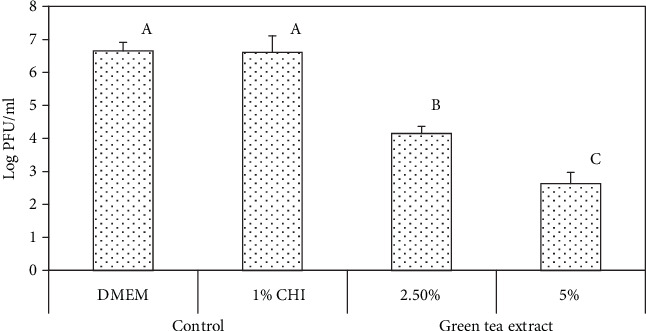
Infectivity of MNV-1 after treatment with chitosan (CHI) film-forming solutions containing green tea extracts and Dulbecco's Modified Eagle Medium (DMEM). Reduction in MNV-1 infectivity was detected by plaque assay after 3 h of incubation at 23 ± 1°C. Error bars indicate standard deviation.

Figure 2 shows that GTE concentrations of 1, 1.5, and 2.5% dissolved in water reduced the MNV-1 plaques by 1.7, 2.5, and 3.3 logs PFU/ml, respectively, after 3 h. The GTE also showed antiviral activity when incorporated into the chitosan film-forming solutions. Chitosan solutions containing 2.5 and 5.0% of the green tea extract resulted in reductions of 2.5 and 4.0 logs PFU/ml, respectively, after 3 h (Figure 3). The chitosan film-forming solutions with no added GTE did not have antiviral activity. The initial MNV-1 concentration was ~10^7^ PFU/ml.

### 3.2. Antiviral Activities of Dried Chitosan Film with the GTE

**Figure 4:**
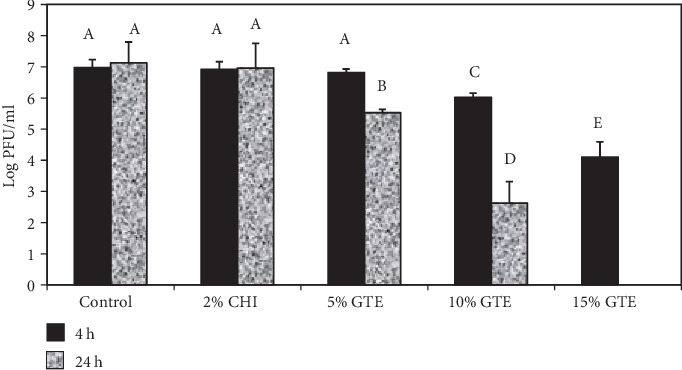
Infectivity of MNV-1 after treatment with chitosan (CHI) films with different levels of green tea extracts. Reduction in MNV-1 infectivity was detected by plaque assay after 4 and 24 h of incubation at 23 ± 1°C. Error bars indicate standard deviation.

Concentrations of 5, 10, and 15% GTE were added during the chitosan film fabrication process, and the antiviral activities of these were evaluated against MNV-1 after 4 and 24 h of incubation. The results are presented in Figure 4. After 4 h of incubation, only the films containing 10 and 15% GTE resulted in statistically significant (*p* < 0.05) reductions of the MNV-1 titer with a decrease of 1.0 and 2.8 logs PFU/ml, respectively. After 24 h of incubation, except for the chitosan-only film, all film types significantly reduced MNV-1 infectivity (*p* < 0.05). Log reductions of 1.6 and 4.5 logs PFU/ml were obtained for the 5 and 10% GTE films, respectively. The film containing 15% GTE reduced the MNV-1 titer to undetectable levels.

### 3.3. Antibacterial Activity of Dried Chitosan Film with GTE

**Figure 5:**
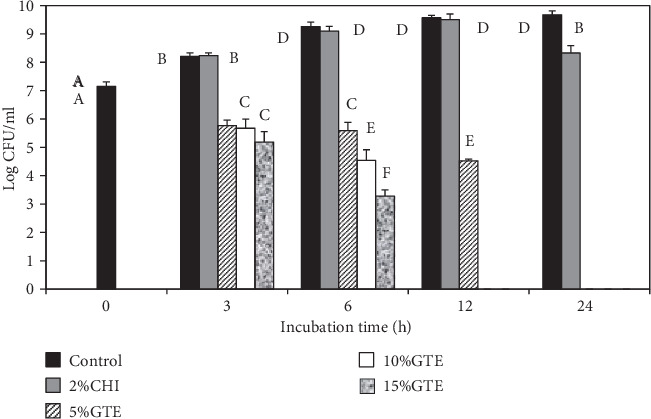
The antibacterial activity of chitosan films incorporated with green tea extract. The effect of green tea extracts incorporated into chitosan films on the survival of *L. innocua* was determined in tryptic soy broth at 37°C. Error bars indicate standard deviation.

**Figure 6:**
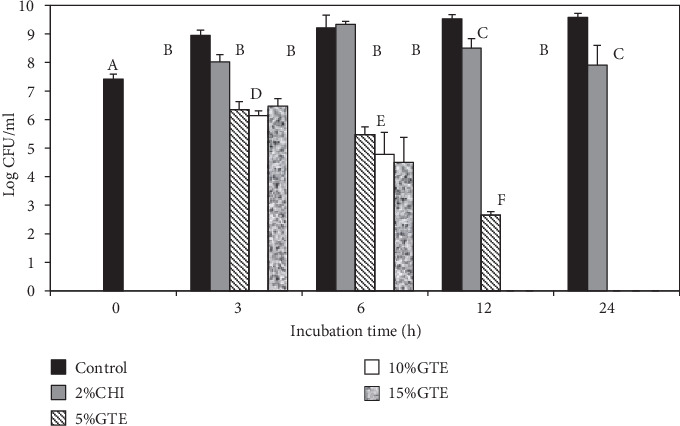
The antibacterial activity of chitosan films incorporated with green tea extract. The effect of green tea extracts (GTE) incorporated into chitosan (CHI) films on the survival of *E. coli* K12 was determined in tryptic soy broth at 37°C. Error bars indicate standard deviation.

The antibacterial activities of the chitosan/GTE films were investigated against the foodborne pathogen surrogates, *E*. *coli* K12 and *L*. *innocua*. The bactericidal activity was enumerated by the viable cell count method to give a quantitative estimation of the efficacy of the films. The inhibitory activities of the GTE-incorporated chitosan-based films are shown in Figures 5 and 6. The samples were enumerated for bacteria viability at 0, 3, 6, 12, and 24 h exposure to the films. Two different controls were tested. These were a control with no added extract and a control with 2% chitosan film (2% CHI) with no added extract.

The bacterial strains used were highly susceptible to the films containing the GTE. The results showed that significant (*p* < 0.05) reductions of both *E*. *coli* K12 and *L*. *innocua* occurred after 3 h of incubation when compared to the controls. However, there were no significant differences (*p* > 0.05) in log reductions between the different GTE films after 3 h of storage. The same was true for *E*. *coli* K12 after 6 h of incubation, where there were no differences between the treatments, even though the GTE films significantly reduced the growth of both bacteria. The GTE films reduced *E*. *coli* K12 and *L*. *innocua* counts by at least 2.0 and 2.5 logs CFU/ml after 3 h of incubation, respectively. At 6 h of incubation, *E*. *coli* K12 reductions of 3.7, 4.4, and 4.7 logs CFU/ml were recorded for the 5, 10, and 15% GTE films, respectively. The 5, 10, and 15% GTE films produced a reduction of 3.7, 4.7, and 6.0 logs CFU/ml in *L. innocua* after 6 h incubation, respectively. After 12 h exposure, the 5% GTE film reduced *E*. *coli* K12 and *L*. *innocua* populations by 5.0 and 6.9 logs CFU/ml, respectively.

All the GTE films (5, 10, and 15%) reduced both *E*. *coli* K12 and *L*. *innocua* counts to undetectable level (<2 logs CFU/ml) after 24 h of contact. However, the 10 and 15% GTE films only needed 12 h to reduce the counts to undetectable levels. In contrast, the chitosan film alone (control) produced significant (*p* < 0.05) log reductions in *E*. *coli* K12 after 12 h exposure. However, reductions for *L*. *innocua* after 12 h were not significant. The reductions for both *E*. *coli* K12 and *L*. *innocua* were only significant after 24 h exposure with reductions of 1.7 and 1.3 logs CFU/ml, respectively.

## 4. Discussion

In designing an antimicrobial polymeric packaging material, the traditional technique is to incorporate an antimicrobial chemical into a polymer having good film-forming properties. The polymer must also have the ability to sequester but subsequently release the antimicrobial agent into the environment containing the microorganisms in order to initialize the killing action. For antimicrobial polymers that are used to coat inanimate substrates such as food preparation surfaces, the preferred method is to use a polymer that sequesters the antimicrobial agent with a high binding affinity. In such cases, microorganisms are killed when they contact the antimicrobial polymer. In this study, the intent was to design the antimicrobial agent for release from the polymer towards the environment with the microorganism. The *E. coli* K12 and *L. innocua* tested in this study were nonpathogenic surrogates for pathogenic *E. coli* O157:H7 and *L. monocytogenes*, respectively. The use of surrogates has been done previously in microbial safety studies at food preparation facilities [20, 21].

In addition to investigating the antiviral and antibacterial activities of GTE dissolved in deionized water (aqueous solutions, DW), we measured these activities in chitosan film-forming solutions (FFS) in order to understand if the film-forming procedure would influence the effectiveness of the GTE. More importantly, the GTE was tested to ascertain if it would retain antimicrobial activity when incorporated into dried chitosan films. Previous studies showed that an antimicrobial agent incorporated into polymeric films or coatings could lose its efficacy, especially if the functional groups responsible for its activity negatively interact with the polymer [11]. Additionally, if the functional groups of the antimicrobial agent bind too tightly to the polymer, the activity of the agent could be reduced [22]. This could occur if the partitioning of the compound to the polymer is too high. The partitioning of a compound describes the level of affinity it has towards one substrate when compared to water. In this present study, the antiviral activity of GTE was retained against MNV-1 when incorporated into the FFS as well as in the resultant dried films. Moreover, the antibacterial activities of the edible films were enhanced against *E*. *coli* K12 and *L*. *innocua* when the GTE was incorporated into the chitosan. This is consistent with the fact that chitosan has antibacterial properties [23].

The pH of the FFS used in this study was determined to be ~4.5 since the ingredients were dissolved in a 1% acetic acid solution. Therefore, one would expect that the acidity can also be the antimicrobial factor. However, some researchers have reported studies showing that most enteric viruses have the ability to survive in acidic conditions [24, 25]. Cannon et al. [26] observed that the infectivity of MNV-1 was minimally affected (less than 1 log reduction of PFU) at pH values of 2, 3, and 4. In another example, a study on foodborne bacteria obtained a sublethal effect (less than 1 log reduction of CFU) when the culture was acidified at pH 4 using acetic acid [27]. In addition to this, since the pH was kept constant in all the test solutions in our study, the antimicrobial effect of the FFS was correlated with the increasing concentrations of the GTE. In future studies, other factors such as pH changes will be considered.

This study investigated the nonenveloped murine norovirus (MNV-1) because it had successfully served as a surrogate for selected foodborne viruses. Using the different levels of GTE, we recorded significant titer reductions of MNV-1. At the same time, chitosan itself did not show noticeable antiviral activity on MNV-1. This agrees with the work of Su and D'Souza [28] who also found that chitosan was ineffective against MNV-1, although it significantly reduced other viral surrogates, including feline calicivirus F-9 (FCV-F9), and bacteriophages. Also, Davis et al. [29] reported significant resistance of MNV-1 to chitosan.

Green tea extract contains catechins such as (−)-epicatechin (EC), (−)-epicatechin gallate (ECG), (−)-epigallocatechin (EGC), and (−)-epigallocatechin gallate (EGCG) [30], with EGCG being the predominant component [31]. The EGCG and the other catechins can be oxidatively coupled to form dimers such as theaflavin-3-gallate (TF-2a), theaflavin-3′-gallate (TF-2b), theasinensin A, P2, and theaflavin-3,3-digallate (TF-3). These polyphenolic catechin compounds, in their pure isolated forms, have been shown to contain varying antiviral efficacies [32, 33]. In a study on nonenveloped viruses, Isaacs et al. [32] investigated the effect of EGCG and EGCG dimers such as theasinensin A, TF-3, and TF-2a. In their study, the theasinensin A reduced the titer of coxsackie A9 virus by about 3.0 logs PFU/ml. However, it was ineffective against other nonenveloped viruses, including coxsackie B4 and echovirus 6. The TF-3, on the other hand, was more effective in inactivating echovirus 6 and coxsackie A9 by 2.0 and 3.5 logs, respectively. At the same time, Isaacs et al. [32] observed that the EGCG itself was not effective in inactivating any virus at any concentration. A recent antiviral study of GTE compounds revealed that EGCG is effective against nonenveloped viruses at only neutral or alkaline pH condition [34]. Therefore, these findings and the acidic conditions in our settings suggest that EGCG may not have been an active antiviral compound in this present study.

Regarding the mechanism of inactivation of the polyphenolic catechins in the GTE, different theories for the results obtained for enveloped and nonenveloped viruses have been proposed [32]. For the nonenveloped viruses, the interaction of the polyphenolic catechins with the viral capsid was responsible for the inactivation results obtained. For the enveloped viruses, the antiviral activity was due to the direct binding of the polyphenolic catechins to the fusion proteins or viral envelope glycoproteins. Additionally, the effect on viral replication has been reported to account for the antiviral activity of polyphenolic compounds [35]. Therefore, the mechanisms responsible for the antiviral activity of GTE on MNV-1 in our study could have ranged from interaction with the virus protein capsid, interference with the viral attachment, to adsorption and/or interference with the viral replication.

Chitosan alone is reported to have antibacterial activities by causing an increase in the cell permeability when it interacts with the negative charges on the surface of the microbial cell. Chitosan also interacts with DNA and messenger RNA and disrupts their normal functions. It also acts as a chelating agent that binds metals and nutrients and makes them unavailable for cell metabolism [36–38]. However, it should be noted that the antimicrobial activity of chitosan can be affected by its degree of deacetylation, molecular weight, and pH of its surroundings [10].

Friedman et al. [39] demonstrated that polyphenols such as (−)-gallocatechin-3-gallate, (−)-epigallocatechin-3-gallate, and theaflavin-3′-gallate in GTE had antimicrobial activity against Gram-positive bacteria. Since then, researchers have tried to incorporate GTE into various edible films and reported on its effectiveness against foodborne bacteria [40, 41]. Theivendran et al. [41] incorporated GTE into soy protein-based films and tested it against *L. monocytogenes* in PBS. The results obtained showed that soy films containing 1% GTE were effective in reducing the *L*. *monocytogenes* counts by about 2.0 logs after 48 h of exposure. In that study, GTE was also combined with nisin, a well-known antibacterial agent against Gram-positive bacteria, and it resulted in an increased antimicrobial activity against the *L. monocytogenes*. Our study found that GTE incorporated into the chitosan was effective against both Gram-negative (*E*. *coli* K12) and Gram-positive (*L*. *innocua*) bacteria. It is generally believed that the impermeability of the outer membrane in the cell wall of Gram-negative bacteria does not allow many types of chemical agents to reach the inner cell membrane [42]. However, in this present study, it appeared that the cell wall structure had little effect on protecting the *E. coli* K12 organism from the antimicrobial compounds.

According to Ultee et al. [43], polyphenols act on the cell membrane of bacteria as the primary target of interaction. The active functional groups in the polyphenols that are responsible for such interaction are the hydroxyls, conjugated double bonds, and galloyl groups. Since the cell membrane keeps the integrity of the cellular structures, damage caused by this interaction is capable of producing a loss of the cell viability. Cell membrane damage also induces the dissipation of the proton motive force (PMF) and/or inhibition of membrane-associated enzyme activity in bacterial cells [44]. EGCG and ECG are reportedly the most effective green tea catechins in terms of antibacterial activity. This was attributed to the presence of the galloyl functional groups in these catechins [44]. As an example, Cox et al. [45] demonstrated the deteriorating effect of these galloyl moieties in catechin on the lipid bilayer membrane of bacterial cells. For our study, the GTE used had a 50% EGCG content and this accounted for the high inactivating capacity of the GTE incorporated into the test films.

## 5. Conclusions

The addition of green tea extract (GTE) into the chitosan-based film-forming solutions and the resultant dried edible films that were produced showed effectiveness in reducing the infectivity of murine norovirus (MNV-1). Also, the films had antibacterial activities against *E. coli* K12 and *Listeria innocua* which served as surrogates of foodborne pathogenic bacteria. This study also showed that the addition of GTE to chitosan did not significantly impede the antimicrobial properties of either compound. Further research is needed in order to investigate the antimicrobial efficacy of the films in actual food systems. Also, analyses on the mechanical, thermal, and sensory properties of the films will be needed before they could be used in commercial applications.

## Figures and Tables

**Table 1:** Formulation of chitosan-based green tea extract (GTE) antimicrobial films.

Antimicrobial films	Chitosan (g)	Glycerol (g)	GTE (g)	Deionized water (ml)
Chitosan only	2	0.6	—	100
5% GTE film	2	3	5	100
10% GTE film	2	5	10	100
15% GTE film	2	7	15	100

## Data Availability

The data used to support this study are available from the corresponding author upon request.
